# The impact of bilberry extract combined with docosahexaenoic acid on the expression of Chrnb4 gene in the sclera of myopic guinea pigs

**DOI:** 10.3389/fmed.2025.1590362

**Published:** 2025-06-04

**Authors:** Mei-hong Zhu, Qian Wen, Tai-nan Lin, Yun Gao, Xiao-ting Liu, Jing-hua Lin, Miao Lin, Qiao-mei Shi

**Affiliations:** ^1^Department of Ophthalmology, Huaqiao University Hospital, Fujian, China; ^2^Department of Ophthalmology, Fujian Provincial Governmental Hospital, Fujian, China

**Keywords:** myopia, scleral remodeling, Chrnb4 gene, bilberry extract, docosahexaenoic acid

## Abstract

**Objective:**

This study aimed to investigate the therapeutic potential of bilberry extract combined with docosahexaenoic acid (DHA) in myopia management by examining their effects on Chrnb4 gene expression in the sclera of lens-induced myopic guinea pigs and elucidating the underlying regulatory mechanisms, thereby providing a theoretical foundation for the development of natural active component-based myopia prevention and treatment strategies.

**Methods:**

One hundred twenty guinea pigs were systematically allocated into five experimental groups: normal control (NC), lens-induced myopia (LIM), LIM+DHA, LIM+bilberry extract (LIM+BE), and LIM+DHA+BE. Myopia was experimentally induced through the application of –6.0D lenses. The intervention groups received daily administrations of DHA (100 mg/kg), bilberry extract (16.5 mg/kg), or their combination for a duration of 12 weeks. Ocular parameters including refractive status and axial length were quantified using a handheld refractometer and A-scan ultrasound, respectively. Choroidal thickness (ChT) and choroidal vascularity index (CVI) were evaluated through swept-source OCT imaging. Molecular analyses encompassing Chrnb4 expression, dopamine concentrations, and TGF-β/MMP-2/TIMP-1 pathway components were conducted using immunofluorescence, ELISA, and Western blot techniques.

**Results:**

Following 4 weeks of myopia induction, the LIM group demonstrated significant myopic changes, including reduced refraction (-3.75 ± 1.35 D), decreased ChT (89.00 ± 10.37 μm), increased axial length (11.33 ± 1.67 mm), and diminished CVI (22.64 ± 4.91%) compared to NC group (all *P* < 0.001). After 12 weeks of therapeutic intervention, the combined treatment group showed marked improvements in ocular parameters, with refraction measuring –2.46 ± 0.92 D and axial length reduced to 9.94 ± 1.10 mm. Notably, ChT and CVI increased by 8.7 and 15.6%, respectively, compared to the LIM group (*P* < 0.05). Molecular analysis revealed significant upregulation of Chrnb4 protein expression, elevation of dopamine levels to 41.13 ± 1.58 nmol/g, suppression of TGF-β and MMP-2 expression, and enhancement of TIMP-1 levels (all *P* < 0.05).

**Conclusion:**

The combination of bilberry extract and DHA demonstrates significant efficacy in controlling myopia progression through multiple mechanisms, including upregulation of Chrnb4 gene expression, modulation of the TGF-β/MMP-2/TIMP-1 signaling pathway, and enhancement of dopamine levels. These findings collectively contribute to the inhibition of scleral remodeling and axial elongation. This study provides valuable insights for the development of novel, multi-targeted natural therapeutic approaches for myopia prevention and treatment.

## 1 Introduction

Myopia, a global ophthalmic disorder, has emerged as a critical public health concern. In recent years (1), its prevalence has risen sharply, particularly among adolescents, posing significant challenges to visual health. The development and progression of myopia not only impair patients’ quality of life but also increase the risk of severe ocular complications, including glaucoma, macular degeneration, and retinal detachment (2). Consequently, in-depth exploration of the pathogenesis and preventive strategies for myopia holds substantial clinical importance.

The onset of myopia is closely linked to ocular growth and development, with scleral remodeling recognized as a pivotal pathological basis for its progression. As a key component of the ocular wall, structural and functional alterations in the sclera directly influence eyeball shape and axial length (3). Studies have demonstrated that in myopic patients, the sclera exhibits disordered collagen fiber arrangement, reduced elastin content, and abnormal extracellular matrix (ECM) remodeling. These changes may enhance scleral extensibility, leading to axial elongation and myopia exacerbation. Thus, elucidating the molecular mechanisms underlying scleral remodeling and their regulatory factors is essential for unraveling the developmental dynamics of myopia (4).

Recent advances in molecular biology have shifted research focus toward the role of specific genes in myopia pathogenesis. Chrnb4 (Choline transporter-like neural substrate family member 4), a potential key gene, is prominently expressed in neural and ocular tissues (5). Emerging evidence suggests that Chrnb4 may regulate ECM remodeling and scleral fibroblast activity, thereby influencing myopia development (6). However, the precise mechanistic role of Chrnb4 in myopic animal models remains poorly understood.

Concurrently, natural bioactive compounds are gaining attention for their therapeutic potential in ophthalmic diseases (7). Bilberry extract, rich in anthocyanins, has demonstrated robust antioxidant and anti-inflammatory properties, offering protective effects on retinal health and visual function (8). Docosahexaenoic acid (DHA), an omega-3 polyunsaturated fatty acid abundant in retinal and neural tissues, is proposed to modulate cellular signaling pathways and inflammatory responses, thereby promoting ocular health. Nevertheless, research on the combined efficacy of bilberry extract and DHA in myopia intervention, particularly their molecular mechanisms, remains limited (9).

This study employs a guinea pig myopia model to investigate the effects of bilberry extract combined with DHA on Chrnb4 expression and its functional implications in scleral remodeling. By analyzing Chrnb4 expression levels under varying intervention conditions and their correlation with scleral structural changes, this research aims to uncover the mechanistic basis of these natural compounds in myopia management. The findings are expected to advance our understanding of myopia pathogenesis and provide a theoretical foundation for developing novel, multi-targeted therapeutic strategies based on natural bioactive components.

## 2 Experimental materials

### 2.1 Experimental animals

A total of 120 healthy male 2-week-old British tricolor short-haired guinea pigs (weight: 110 ± 10 g) were purchased from Jiangxi Zhonghong Boyuan Biotechnology Co., Ltd. The animals were housed at the Animal Experiment Center of the Fujian Provincial Center for Disease Control and Prevention. Guinea pigs were maintained in transparent plastic cages (4–5 animals per cage) under controlled conditions: illumination intensity of approximately 300 lux, room temperature of 22°C, ad libitum access to food and water, and a natural 12-h light/dark cycle. Animal management strictly adhered to the *Animal Experiment Regulations of the Ministry of Health of the People’s Republic of China*, and the experimental protocol was approved by the Ethics Committee of Fujian Provincial Government Hospital (Approval No.: RL2024-07).

### 2.2 Animal grouping

Prior to grouping, all animals underwent ocular examinations by experienced optometrists using a computerized refractometer and handheld retinal camera to exclude congenital myopia, cataracts, or corneal diseases. The 120 guinea pigs were randomly divided into five groups (24 animals per group): Normal control (NC) group; Lens-induced myopia (LIM) group; LIM + DHA + bilberry extract (LIM+DHA+BE) group; LIM + DHA group; LIM + bilberry extract (LIM+BE) group.

### 2.3 Defocus-induced myopia model establishment

A defocus-induced myopia model was established using custom-made –6.0D polymethyl methacrylate (PMMA) lenses. The right eye was fitted with a –6.0D lens, while the left eye served as an untreated control. Lenses were cleaned daily to ensure transparency. Except for the NC group, all other groups wore 3D-printed head-mounted devices with PMMA lenses fixed on the right eye for 4 weeks to induce defocus myopia. Successful model establishment was confirmed by increased myopic refractive error and axial elongation in the right eye post-modeling.

### 2.4 Preparation of DHA and bilberry extract

Pharmacological equivalent dose conversion based on body surface area normalization (BSA): Convert the human recommended dose to the equivalent dose in guinea pigs using the following formula:Animal dose (mg/kg) = Human dose (mg/kg) × (Animal average body weight (kg) / Human average body weight (kg)) × 0.33 × Safety factor (10).

The human average body weight is assumed to be 60 kg, and the guinea pig average body weight is assumed to be 0.3 kg. The safety factor is set at 5 times to cover interspecies differences and potential metabolic variations. For DHA: The human recommended dose is 4 mg/kg → The equivalent dose in guinea pigs is 20 mg/kg. For anthocyanin from bilberry extract: The human recommended dose is 0.66 mg/kg → The equivalent dose in guinea pigs is 3.3 mg/kg.

#### 2.4.1 DHA

DHA algal oil (35% DHA content) was administered via oral gavage at a dose of 100 mg/kg (five times the human recommended daily intake of 20 mg/kg) (11).

#### 2.4.2 Bilberry extract

Bilberry extract powder (70% ethanol extraction) was dissolved in edible vegetable oil and administered at a dose of 16.5 mg/kg (five times the human recommended daily intake of 3.3 mg/kg) (12).

#### 2.4.3 Combined intervention

The mixture contained DHA algal oil (100 mg/kg) and bilberry extract (16.5 mg/kg). Due to its viscous consistency, the mixture was diluted with edible oil before administration.

## 3 Experimental content

### 3.1 Application of handheld refractometer in refractive measurement

Before the experiment, and at 2 weeks and 4 weeks after modeling, refractive measurements were conducted on experimental guinea pigs using a handheld refractometer. Prior to testing, 1% cyclopentolate hydrochloride eye drops were used for pupil dilation, with 1 drop administered twice, 10 minutes apart. After waiting for 30 minutes, refractive measurements were taken on both eyes. The entire measurement process was carried out by an experienced optometrist in a darkroom environment.

### 3.2 Scan for axial length measurement

Axial length was measured using an A-scan ultrasound (Tianjin Suwei). Prior to measurement, 0.5% proparacaine hydrochloride was used for surface anesthesia of the eye. Instrument parameters were set as follows: the velocity of sound in the anterior chamber was 1557 m/s, in the vitreous body was 1540 m/s, and in the lens was 1723 m/s. During measurement, the probe was lightly touched to the corneal surface, placed perpendicular to the corneal apex, and 10 consecutive measurements were manually taken, discarding clearly erroneous values. The average value was used, and the entire procedure was completed by the same skilled technician.

### 3.3 Measurement of choroidal thickness and choroidal vascular index

The guinea pig ChBP (Choroidal Blood Perfusion) was measured using the TUPAI Beiming-Kun High-definition Full-field Sweep OCTA, BM-400K (BMizar, TowardPi Medical Technology, Beijing, China). Prior to the measurement, 1% cyclopentolate hydrochloride was used for pupil dilation, and the guinea pigs were anesthetized with gas anesthesia. The OCTA mode was selected for blood flow acquisition. The guinea pig was placed on a small platform in front of the probe, and any moisture on the corneal surface was removed with a clean cotton swab. The probe was aligned with the guinea pig’s eye. The scanning system was moved forward using the mouse scroll wheel until the optic disc appeared centrally on the fundus image. This provided a single B-scan image. The OCT B-scan image was automatically centered and positioned for optimal signal strength. Horizontal and vertical scans were performed around the optic disc, with a scanning range of 12x12mm, and each position was scanned twice for the best image quality. The OCTA image quality was considered acceptable if the score was ≥ 6. The analysis software was then used to assess choroidal tissue thickness and blood flow.

### 3.4 Immunofluorescence detection: immunofluorescence detection of Chrnb4 protein expression in scleral fibroblasts

#### 3.4.1 Isolation, culture, and modeling of guinea pig scleral fibroblasts

**FIGURE 1:**
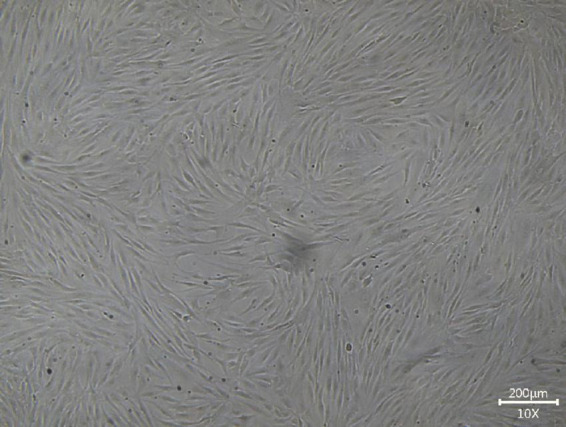
Cultivation of guinea pig scleral fibroblasts.

Scleral tissue collection: After euthanasia, eyeballs were rapidly enucleated and immersed in pre-cooled PBS (containing antibiotics) to remove blood. The dense white scleral tissue was retained. The sclera was cut into 1–2 mm^2^ pieces and repeatedly rinsed with PBS until pigment-free. Collagenase digestion: Scleral fragments were transferred to a centrifuge tube containing 1–2 mg/mL collagenase and digested at 37°C for 1–2 h (adjusted based on tissue volume). Gentle pipetting was performed every 15 min to release cells. The reaction was terminated by adding serum-containing medium, followed by centrifugation (1,000 rpm, 5 min) and supernatant removal. The cell suspension was filtered through a cell strainer, resuspended in complete medium, and seeded into culture flasks. Cells were incubated at 37°C with 5% CO_2_. After 24 h, the medium was replaced to remove non-adherent cells and debris, with subsequent medium changes every 2–3 days. At 80–90% confluency, cells were passaged. Culture supernatant was discarded, and cells were washed twice with PBS, digested with 0.25% trypsin (containing 0.02% EDTA), and neutralized with medium. The cell suspension was centrifuged (1m000 rpm, 3 min), resuspended, and split into new flasks at a 1:3 ratio. Fibroblasts exhibited a spindle-shaped or irregular stellate morphology. Cells in 96-well plates were incubated with 10 μL CCK-8 reagent per well for 2 h. Absorbance was measured at 450 nm using a microplate reader. Modeling: Cells were cultured under hypoxia (HO, 1% O_2_) for 48 h to establish the disease model, with normoxia (NO, 20% O_2_) as the control. Model validation: qPCR confirmed increased α-SMA and decreased COL1A1 expression in the hypoxia group (Figure 1).

#### 3.4.2 Cranberry extract and DHA concentration screening

Cell treatment: Scleral fibroblasts were treated with cranberry extract (0, 12, 24, 36, 48, 60 μg/mL) or DHA (0, 10, 20, 30, 40, 50 μmol/L) for 48 h. Cell viability was assessed via CCK-8 (Figure 1). Optimal concentrations were determined as 60 μg/mL cranberry extract and 50 μmol/L DHA. Experimental groups: 1. Blank control 2. Hypoxia model (HO) 3. HO + DHA (50 μmol/L) 4. HO + cranberry extract (60 μg/mL) 5. HO + DHA + cranberry extract (combination).

#### 3.4.3 Immunofluorescence staining

Cell fixation: Cells were washed 3 × with PBS (3 min each), fixed with 4% paraformaldehyde (15 min), and rinsed again.

Blocking: After PBS washes, non-specific binding was blocked with 5% BSA (37°C, 30 min).

Primary antibody incubation: Anti-Chrnb4 (1:200 dilution) was added and incubated overnight at 4°C. Secondary antibody incubation: Cells were rewarmed (45 min, RT), washed, and incubated with CY3-conjugated secondary antibody (1:200, 37°C, 35 min). Counterstaining and mounting: Nuclei were stained with DAPI (2 min, dark), excess dye was removed with PBS, and cells were mounted with 50% glycerol. Imaging: Fluorescence microscopy was performed (DAPI: excitation 330–380 nm/emission 420 nm, blue; CY3: excitation 510–560 nm/emission 590 nm, red).

#### 3.4.4 Data analysis fluorescence intensity

Quantified using ImageJ to evaluate Chrnb4 protein expression. Interpretation: Nuclei (blue) were visualized under UV light; Chrnb4-positive signals (red) indicated protein localization.

### 3.5 Enzyme-linked immunosorbent assay (ELISA) for dopamine

The guinea pig scleral tissue was quickly frozen in liquid nitrogen or stored at –80°C. The tissue was homogenized in pre-chilled PBS or lysis buffer, then centrifuged (12,000 rpm, 10 min, 4°C) and the supernatant was collected. BCA method was used to measure protein concentration for normalization. In the homogenized tissue, 0.1 M perchloric acid (HClO_4_) or 0.1 M hydrochloric acid (HCl) was added, centrifuged, and the supernatant neutralized to pH 7.0. Solid-phase extraction columns were used to purify dopamine, removing impurities. A specific ELISA kit for dopamine (e.g., competitive ELISA kit) was used. Dopamine standards were prepared at concentrations of 50 pg/mL. The standards and samples were added to pre-coated dopamine antibody plates. Biotinylated dopamine was added and incubated at room temperature for 1–2 h. The plate was washed 3–5 times to remove unbound material. HRP-labeled streptavidin was added and incubated at room temperature for 30 min. After another washing, TMB substrate was added, and the plate was incubated for 15–30 min at room temperature in the dark. The reaction was stopped with a stop solution, and the color changed from blue to yellow. The absorbance (OD value) at 450 nm was measured using an enzyme marker. A standard curve was drawn with dopamine concentration on the x-axis and OD value on the y-axis. The dopamine concentration in samples was calculated from the standard curve. The dopamine content per unit of protein was expressed as pg/mg protein.

### 3.6 Western blot detection of TGF-β, MMP-2, TIMP-1

The Western Blot detection process for TGF-β, MMP-2, and TIMP-1 in guinea pig scleral tissue: The scleral tissue was placed in lysis buffer containing protease inhibitors and homogenized or sonicated to release total proteins. After centrifugation (12,000 rpm, 15 min, 4°C), the supernatant was collected and protein concentration was measured using the Bradford method. The separation gel was prepared according to the molecular weight of the target protein: TGF-β1 (∼25 kDa) with 8–12% gel, MMP-2 (72 kDa) and MMP-9 (92 kDa) with 10% gel, and TIMP-1 (28 kDa) with 10–12% gel. After loading the samples, gel electrophoresis was performed at a constant voltage (80–120 V) until the bromophenol blue marker reached the bottom of the gel. The proteins were transferred to PVDF membranes via wet transfer. The membranes were blocked with 5% skim milk or 3% BSA for 1 h at room temperature to block non-specific binding sites. The membranes were incubated with specific primary antibodies for each target protein, followed by HRP-conjugated secondary antibodies. ECL chemiluminescence reagents were used for signal detection, and exposure was optimized to avoid signal saturation. The internal reference proteins (e.g., β-actin or GAPDH) were tested in parallel to ensure uniformity in sample loading. ImageJ software was used to analyze the grayscale values of the target bands and the reference, and the relative expression levels were calculated (target protein grayscale value/reference protein grayscale value).

### 3.7 Statistical analysis

GraphPad Prism 9.0 software was used for graphical plotting and statistical analysis. All experiments were repeated three times, and the quantitative results were expressed as the mean ± standard deviation (X ± S). One-way ANOVA was used for comparisons among multiple groups, and pairwise comparisons were conducted using the S-N-K method. A *P* value of < 0.05 was considered statistically significant.

## 4 Results

### 4.1 Changes in refractive power, axial length, and intraocular pressure

#### 4.1.1 Changes in refractive power and axial length

**FIGURE 2:**
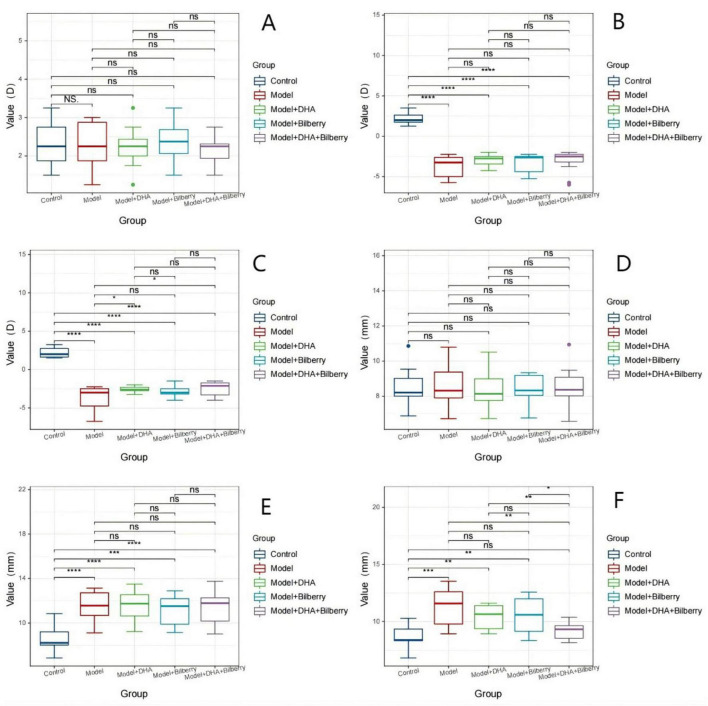
Comparison of optometry and axial length among different groups FIG3. **(A)** Comparison of refractive error results among groups before modeling. **(B)** Comparison of axial length results among groups before modeling. **(C)** Comparison of refractive error results among groups 4 weeks after modeling. **(D)** Comparison of axial length results among groups 4 weeks after modeling; **(E)** Comparison of refractive error results among groups 8 weeks after treatment. **(F)** Comparison of axial length results among groups 8 weeks after treatment. LIM: Lens-induced myopia; PMMA: Polymethyl methacrylate; NC: Normal control; LIM: Lens-induced myopia; DHA: Docosahexaenoic acid; BE: Bilberry extract. ns *p* > 0.05, *p < 0.05, **p < 0.01, ***p < 0.001, ****p < 0.0001.

Before modeling, there were no statistically significant differences in the refractive power and axial length of the right eye among the groups of guinea pigs. The refractive power (D) for the right eye was as follows: Control group (2.32 ± 0.57D), Model group (2.39 ± 0.53D), Model+DHA group (2.25 ± 0.49D), Bilberry group (2.20 ± 0.55D), and DHA+Bilberry group (2.21 ± 0.44D) (Figure 2A). The axial length (mm) for the right eye was as follows: Control group (8.55 ± 1.07 mm), Model group (8.58 ± 1.07 mm), Model+DHA group (8.53 ± 1.04 mm), Bilberry group (8.55 ± 1.16 mm), and DHA+Bilberry group (8.55 ± 1.16 mm) (Figure 2B).

Four weeks after modeling, compared with the Control group (right eye refractive power 2.27 ± 0.58D), the refractive power in the Model group (-3.75 ± 1.35D), Model+DHA group (-3.50 ± 1.09D), Bilberry group (-3.55 ± 1.05D), and DHA+Bilberry group (ns *p* > 0.05, *p < 0.05, **p < 0.01, ***p < 0.001, ****p < 0.0001.3.16 ± 1.25D) all showed significant myopic changes (*P* < 0.01) (Figure 2C). Similarly, compared to the Control group (right eye axial length 8.91 ± 1.16 mm), the axial lengths in the Model group (11.33 ± 1.67 mm), Model+DHA group (11.51 ± 1.40 mm), Bilberry group (11.09 ± 1.59 mm), and DHA+Bilberry group (11.27 ± 1.58 mm) were all significantly longer (*P* < 0.01) (Figures 2D).

Twelve weeks after modeling, compared with the Model group (right eye refractive power –3.75 ± 1.55D), the refractive power in the Model+DHA group (-2.58 ± 0.41D), Bilberry group (-2.87 ± 0.79D), and DHA+Bilberry group (-2.46 ± 0.92D) all showed a reduction (*P* < 0.05) (Figure 2E). Compared with the Model group (right eye axial length 11.31 ± 1.7 0 mm), the axial lengths in the Model+DHA group (10.41 ± 1.06 mm), Bilberry group (10.59 ± 1.56 mm), and DHA+Bilberry group (9.94 ± 1.10 mm) were significantly shorter (*P* < 0.05) (Figure 2F).

(A) Comparison of refractive error results among groups before modeling; (B) Comparison of axial length results among groups before modeling; (C) Comparison of refractive error results among groups 4 weeks after modeling; (D) Comparison of axial length results among groups 4 weeks after modeling; (3) Comparison of refractive error results among groups 8 weeks after treatment; (F) Comparison of axial length results among groups 8 weeks after treatment. LIM: Lens-induced myopia; PMMA: Polymethyl methacrylate; NC: Normal control; LIM: Lens-induced myopia; DHA: Docosahexaenoic acid; BE: Bilberry extract. ns *p* > 0.05, *p < 0.05, **p < 0.01, ***p < 0.001, ****p < 0.0001.

### 4.2 Changes in choroidal thickness (ChT) and choroidal vascular index (CVI)

Before the experiment, there were no statistically significant differences in choroidal thickness (ChT) among the groups. The ChT (μm) was as follows: Control group (108.82 ± 14.86μm), Model group (107.18 ± 12.42 μm), Model+DHA group (104.6 ± 8.29μm), Bilberry group (108.5 ± 10.76 μm), and DHA+Bilberry group (105 ± 11.31 μm) (Figure 4A). Four weeks after the experiment, compared with the Control group (ChT 108.18 ± 14.55 μm), the ChT in the Model group (89.00 ± 10.37 μm), Model+DHA group (86.80 ± 8.38 μm), Bilberry group (89.80 ± 8.57 μm), and DHA+Bilberry group (87.58 ± 9.86 μm) all showed significant thinning (*P* < 0.001) (Figure 4B). Twelve weeks after the experiment, compared with the Model group (ChT 89.91 ± 10.86 μm), the choroidal thickness in the DHA+Bilberry group (97.67 ± 10.29 μm) and Model+DHA group (93.9 ± 10.09 μm) increased (*P* < 0.001; *P* < 0.05). The increase in the DHA+Bilberry group was particularly significant, and the difference with the Model+DHA group and Bilberry group (97.30 ± 9.52 μm) was statistically significant (*P* < 0.01; *P* < 0.01) (Figure 4C). The increase in ChT in the Bilberry group compared to the Model group was not statistically significant.

Before the experiment, there were no significant differences in the Choroidal Vascular Index (CVI) among the groups. The CVI (%) was as follows: Control group (31.02 ± 6.53%), Model group (30.91 ± 6.61%), Model+DHA group (34.10 ± 5.82%), Bilberry group (29.6 ± 5.28%), and DHA+Bilberry group (31.50 ± 6.59%) (Figure 4D).

Four weeks after the experiment, compared with the Control group (CVI 30.55 ± 5.85%), the CVI in the Model group (22.64 ± 4.91%), Model+DHA group (18.4 ± 6.8%), Bilberry group (20.8 ± 4.08%), and DHA+Bilberry group (20.83 ± 3.19%) all showed significant reductions (*P* < 0.001) (Figure 4E). Twelve weeks after the experiment, compared with the Model group (CVI 22.64 ± 4.43%), the CVI in the DHA+Bilberry group (26.17 ± 5.52%) and Model+DHA group (23.9 ± 3.54%) increased (*P* < 0.001; *P* < 0.05). The increase in CVI in the DHA+Bilberry group was particularly significant, and the difference with the Model+DHA group and Bilberry group (23.7 ± 3.89%) was statistically significant (*P* < 0.01; *P* < 0.001) (Figure 4F). There was no statistically significant difference in CVI between the Model+Bilberry group and the Model group.

**FIGURE 3:**
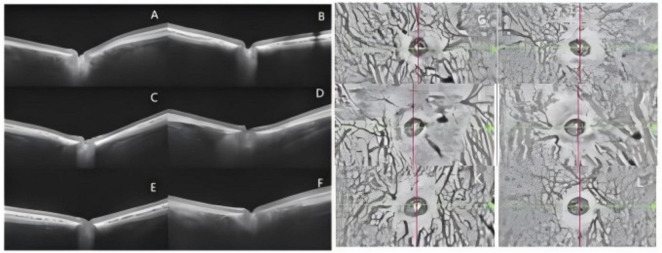
Comparison of choroidal thickness and CVI among groups. **(A)** Right-eye ChT of the Control group; **(B)** Right-eye ChT of the Model group at 4 weeks after modeling; **(C)** Right-eye ChT of the Model group after 8 weeks of treatment; **(D)** Right-eye ChT of the Model+DHA group after 8 weeks of treatment; **(E)** Right-eye ChT of the Bilberry group after 8 weeks of treatment; **(F)**. Right-eye ChT of the DHA+Bilberry group after 8 weeks of treatment; **(G)** Right-eye CVI of the Control group;(H) Right-eye CVI of the Model group at 4 weeks after modeling; **(I)** Right-eye CVI of the Model group after 8 weeks of treatment; **(J)** Right-eye CVI of the Model+DHA group after 8 weeks of treatment; **(K)** Right-eye CVI of the Bilberry group after 8 weeks of treatment; **(L)** Right-eye CVI of the DHA+Bilberry group after 8 weeks of treatment.

**FIGURE 4:**
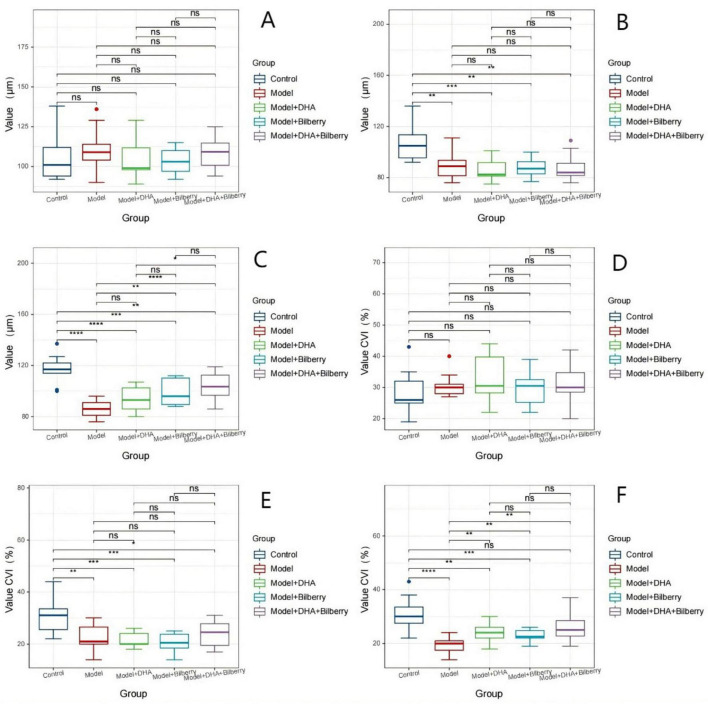
Comparison of choroidal thickness and CVI data differences among groups. Comparison of ChT and CVI results in guinea pigs. **(A)** Comparison of ChT across groups before modeling. **(B)** Comparison of ChT across groups 4 weeks after modeling. **(C)** Comparison of ChT across groups 8 weeks after treatment. **(D)** Comparison of CVI across groups before modeling. **(E)** Comparison of CVI across groups 4 weeks after modeling. **(F)** Comparison of CVI across groups 8 weeks after treatment. ChT: Choroidal thickness; CVI: Choroidal vascularity index. ns *p* > 0.05, *p < 0.05, **p < 0.01, ***p < 0.001, ****p < 0.0001.

The changes in choroidal thickness (ChT) and choroidal vascular index (CVI) images and statistical results are shown in Figures 3, 4.

### 4.3 immunofluorescence detection of related protein expression in tissues

**FIGURE 5:**
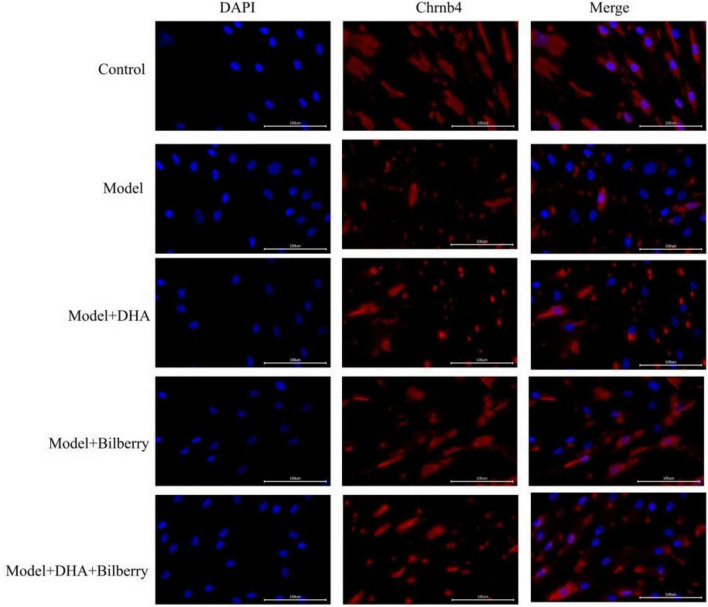
Immunofluorescence results of scleral fibroblasts in each group.

**FIGURE 6:**
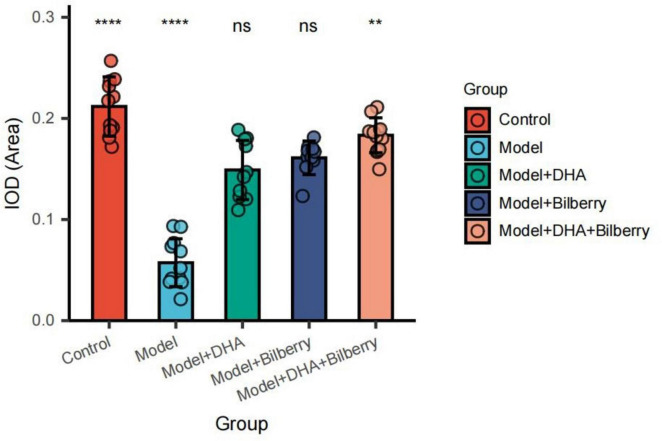
Comparison of immunofluorescence results among groups. ns *p* > 0.05, *p < 0.05, **p < 0.01, ***p < 0.001, ****p < 0.0001.

Compared to the Control group, the expression of Chrnb4 protein in the Model group was significantly reduced. In the Model+DHA, Model+Bilberry, and Model+DHA+Bilberry groups, the expression of Chrnb4 protein was significantly increased compared to the Model group (Figures 5, 6).

### 4.4 comparison of dopamine levels among different groups of guinea pigs

In this experiment, significant differences in dopamine levels (unit: nmol/g) were observed among different groups. The specific results are as follows:

Control group: Dopamine level was 48.57 ± 1.76 nmol/g, the highest among all groups, indicating that dopamine levels are maintained at a relatively high level under normal conditions. Model group: Dopamine level was 17.15 ± 1.80 nmol/g, significantly lower than the Control group, indicating that dopamine levels were markedly suppressed in the model group. Model+Bilberry group: Dopamine level was 33.20 ± 1.36 nmol/g, higher than the Model group but still lower than the Control group. Model+DHA group: Dopamine level was 36.63 ± 1.06 nmol/g, also higher than the Model group but lower than the Control group, suggesting that DHA has a certain enhancing effect on dopamine levels. Model+DHA+Bilberry group: Dopamine level was 41.13 ± 1.58 nmol/g, the highest among all intervention groups but still lower than the Control group. This indicates that the combination of DHA and bilberry extract has a more pronounced effect on restoring dopamine levels compared to either intervention alone.

#### 4.4.1 Statistical analysis results

The *F*-value was 50.797, indicating that the differences in dopamine levels among the groups were highly statistically significant. The *P*-value was less than 0.001, further confirming that these differences were significant. This suggests that different interventions have distinct effects on dopamine levels.

In summary, both DHA and bilberry extract, either alone or in combination, significantly increased dopamine levels in the model group. However, the combined use of DHA and bilberry extract showed the best effect on restoring dopamine levels, although it did not fully reach the normal level (Table 1).

**TABLE 1:** Comparison of dopamine content in each group of guinea pigs.

Group	n	Dopamine (nmol/g)
Control group	11	48.57 ± 1.76
Model group	11	17.15 ± 1.80
Model+bilberry group	10	33.20 ± 1.36
Model+DHA group	10	36.63 ± 1.06
Model+DHA+bilberry group	12	41.13 ± 1.58
*F*-value		50.797
*P*-value		<0.001

### 4.5 WB detection of TGF-β, MMP-2, and TIMP-1 proteins

**FIGURE 7:**
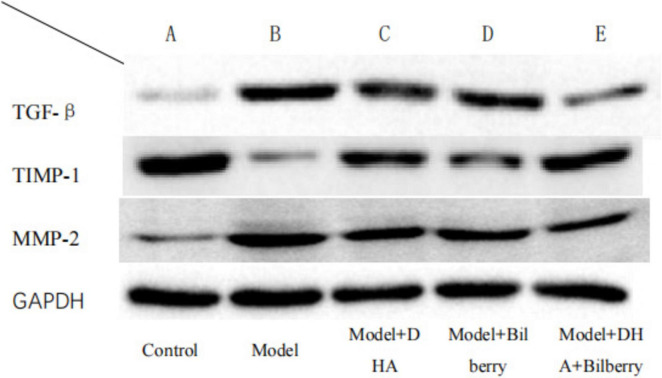
WB detection results of each group.

**FIGURE 8:**
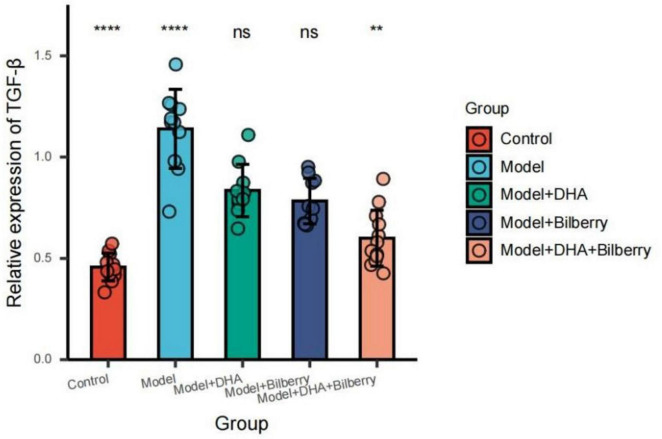
Comparison of TGF-β results among different groups. ns *p* > 0.05, *p < 0.05, **p < 0.01, ***p < 0.001, ****p < 0.0001.

As shown in the results of Figure 7, compared with the Control group, the expression of TGF-β protein in the Model group was significantly increased. Compared with the Model group, the expression of TGF-β protein in the Model+DHA, Model+Bilberry, and Model+DHA+Bilberry groups was significantly reduced (Figure 8).

**FIGURE 9:**
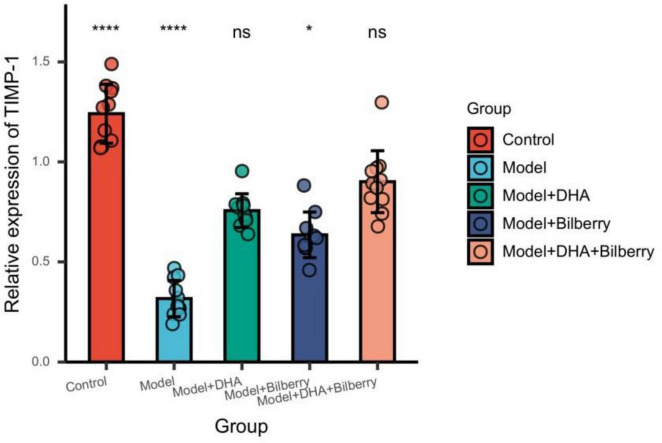
Comparison of TIMP-1 results among different groups. ns *p* > 0.05, *p < 0.05, **p < 0.01, ***p < 0.001, ****p < 0.0001.

Compared with the Control group, the expression of TIMP-1 protein in the Model group was significantly reduced. Compared with the Model group, the expression of TIMP-1 protein in the Model+DHA, Model+Bilberry, and Model+DHA+Bilberry groups was significantly increased (Figure 9).

**FIGURE 10:**
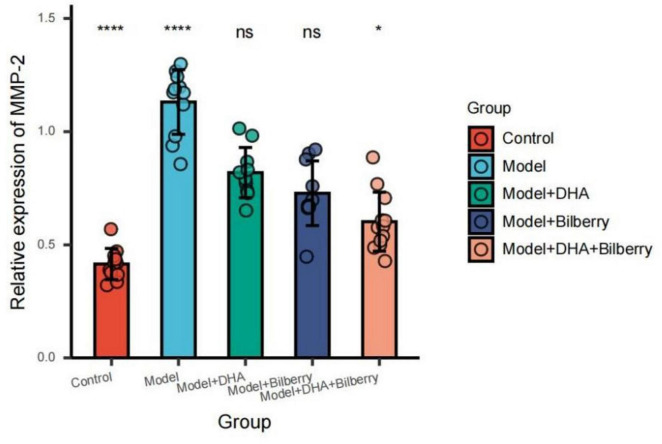
Comparison of MMP-2 results among groups. ns *p* > 0.05, *p < 0.05, **p < 0.01, ***p < 0.001, ****p < 0.0001.

Compared with the Control group, the expression of MMP-2 protein in the Model group was significantly increased. Compared with the Model group, the expression of MMP-2 protein in the Model+DHA, Model+Bilberry, and Model+DHA+Bilberry groups was significantly reduced (Figure 10).

## 5 Discussion

This study investigated the combined effect of bilberry extract and docosahexaenoic acid (DHA) on the expression of the Chrnb4 gene in the sclera of guinea pigs with lens-induced myopia (LIM). The results demonstrate that both bilberry extract and DHA, either alone or in combination, significantly influenced the progression of myopia by modulating various ocular parameters and molecular pathways, particularly the Chrnb4 gene expression, dopamine levels, and the TGF-β/MMP-2/TIMP-1 signaling pathway.

### 5.1 Effect of DHA and bilberry extract on ocular parameters

One of the key findings of this study is that the combination of bilberry extract and DHA notably improved the refractive status and axial length of the eyes compared to the model group. After 12 weeks of treatment, the combined intervention group showed significant improvement in refractive power (–2.46 ± 0.92 D) and axial length (9.94 ± 1.10 mm), compared to the model group, where refractive power was –3.75 ± 1.35 D and axial length was 11.31 ± 1.70 mm. This indicates that the combination treatment was effective in mitigating the progression of myopia, which is consistent with the hypothesis that DHA and bilberry extract, through their combined mechanisms, can retard ocular elongation and reduce the severity of myopia (13–15).

In recent years, the clinical significance of choroidal thickness (Choroidal Thickness, ChT) has increasingly gained attention (16), as it can intuitively reflect the changes in the choroid associated with myopia. With technological advancements, the choroidal vascularity index (Choroidal Vascularity Index, CVI) has emerged as a more reliable indicator for assessing the vascular structure of the choroid (17). CVI is defined as the ratio of the choroidal lumen area to the total choroidal area. It can describe the relative changes in the choroidal vasculature and stroma, allowing for a more accurate assessment of the structural changes in the choroid caused by myopia (18). Furthermore, the choroidal thickness (ChT) and choroidal vascularity index (CVI) were also positively affected by the combined treatment. After 12 weeks, ChT was significantly increased in the DHA+Bilberry group (97.67 ± 10.29 μm) compared to the model group (89.91 ± 10.86 μm), showing a recovery in the choroidal structure. Similarly, the CVI in the combined treatment group increased to 26.17 ± 5.52%, compared to the model group’s 22.64 ± 4.43%, which indicates an enhancement in choroidal blood flow. This recovery in both structural and functional aspects of the choroid suggests that bilberry extract and DHA work synergistically to improve ocular health and counteract the adverse effects of myopia (19).

### 5.2 Molecular mechanisms: Chrnb4, dopamine, and the TGF-β/MMP-2/TIMP-1 pathway

The Chrnb4 gene, also known as the choline transporter-like neural substrate family member 4, is actually more accurately referred to as the Cholinergic Receptor Nicotinic Beta 4 Subunit. This gene encodes a subunit of the nicotinic acetylcholine receptor, which is primarily distributed in the nervous system and muscle tissues (20). It plays a crucial role in synaptic neurotransmission between neurons or between neurons and muscle cells.

In the context of eye health and myopia, research has indicated that the Chrnb4 gene is highly expressed in neural and ocular tissues. It is believed that Chrnb4 may influence the development of myopia by regulating the remodeling of the extracellular matrix (ECM) and the activity of scleral fibroblasts (21). The ECM is a critical component of the sclera, and its structural integrity is essential for maintaining the shape and function of the eye. Changes in ECM remodeling can affect the biomechanical properties of the sclera, potentially leading to alterations in eye shape and the progression of myopia (22).

While the exact mechanisms through which Chrnb4 influences myopia are still being explored, its role in modulating the ECM and scleral fibroblast activity suggests that it could be a significant factor in the pathogenesis of this condition. Further research is needed to elucidate the precise pathways and interactions involving Chrnb4 in the development and progression of myopia.

The molecular analysis further highlights the potential mechanisms behind the protective effects of DHA and bilberry extract on myopia progression. Chrnb4 expression, which is involved in scleral remodeling and may affect myopia development, was significantly upregulated in the combined treatment groups (Model+DHA+Bilberry) compared to the model group. This suggests that Chrnb4 plays an important role in the protective effects of DHA and bilberry extract, possibly by influencing extracellular matrix (ECM) remodeling in the sclera, which is a key factor in axial elongation during myopia development.

Dopamine, a crucial neurotransmitter known for its inhibitory role in the development of myopia (23), was also significantly increased in the combined treatment group. The dopamine content reached 41.13 ± 1.58 nmol/g in the DHA+Bilberry group, compared to the model group’s 17.15 ± 1.80 nmol/g. This finding suggests that both DHA and bilberry extract have dopamine-enhancing effects, which likely contribute to their ability to regulate scleral remodeling and reduce axial elongation. Given that lower dopamine levels have been associated with myopia progression, the restoration of dopamine levels by these natural compounds may provide an important mechanism for myopia control.

The results of TGF-β, MMP-2, and TIMP-1 expression levels further support this conclusion (24). TGF-β, a critical regulator of ECM production, was significantly downregulated in the DHA, bilberry extract, and combined intervention groups compared to the model group, indicating a suppression of scleral remodeling. Similarly, MMP-2, an enzyme involved in the breakdown of ECM, was significantly reduced, while TIMP-1, a tissue inhibitor of metalloproteinases, was significantly upregulated in the intervention groups. These findings suggest that the DHA and bilberry extract combination helps maintain a balanced ECM environment by modulating the TGF-β/MMP-2/TIMP-1 pathway, which is crucial in regulating scleral structure and preventing excessive axial elongation.

**FIGURE 11:**
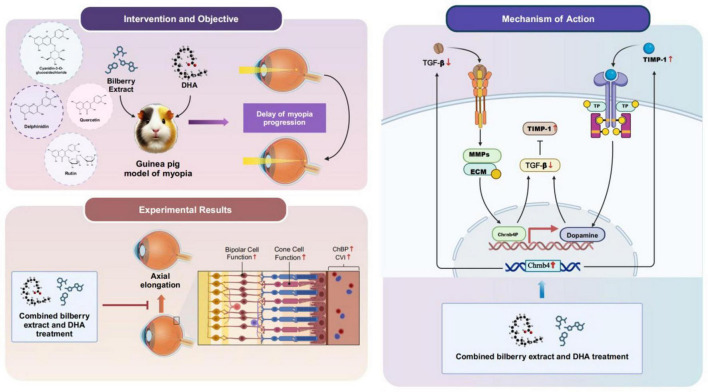
Mechanism diagram of bilberry extract combined with DHA for myopia treatment.

Proposed Mechanism of Chrnb4 in Regulating Myopia via Dopaminergic and TGF-β/MMP-2/TIMP-1 Pathways The mechanism diagram is shown in Figure 11.

Based on the findings, we propose that Chrnb4 modulates myopia progression through a multi-layered signaling network involving dopamine and the TGF-β/MMP-2/TIMP-1 axis, as outlined below (see schematic framework): Functional Localization and Upstream Regulation of Chrnb4 encodes the β4 subunit of the nicotinic acetylcholine receptor (nAChR), predominantly expressed in retinal amacrine cells, bipolar cells, and scleral fibroblasts. Retinal cholinergic interneurons release acetylcholine (ACh), which activates nAChR containing the β4 subunit, triggering Ca^2+^ influx and cAMP/PKA signaling cascades. nAChRβ4 activation stimulates dopamine release from retinal dopaminergic amacrine cells via Ca^2+^-dependent synaptic vesicle exocytosis. Dopaminergic Signaling and Axial Growth Inhibition. Dopamine binds to D1/D2 receptors, activating adenylate cyclase to elevate intracellular cAMP levels, thereby suppressing pro-axial elongation signals. Light adaptation integration: Chrnb4-mediated cholinergic signaling enhances photic adaptation through the retinal ON pathway, indirectly sustaining rhythmic dopamine secretion to counteract myopia progression. Dopamine-D2 receptor signaling inhibits ERK1/2 phosphorylation, blocking TGF-β/Smad-driven transcriptional activation of MMP-2. ECM Metabolic Balance Regulation. MMP-2 suppression: Elevated dopamine inhibits NF-κB nuclear translocation via the D1 receptor/cAMP/PKA pathway, reducing MMP-2 gene transcription. TIMP-1 upregulation: TGF-β1 promotes TIMP-1 expression through Smad4-dependent pathways. Conversely, Chrnb4 deficiency increases histone H3K27me3 modification at the TIMP-1 promoter, repressing its transcription. Scleral Remodeling and Biomechanical Changes. Loss of Chrnb4 leads to hyperactivation of the TGF-β1/Smad3 pathway, driving scleral fibroblast-to-myofibroblast differentiation with increased α-SMA expression and enhanced collagen contraction. An elevated MMP-2/TIMP-1 ratio accelerates degradation of type I/III collagen, reducing scleral biomechanical strength and promoting axial elongation.

The combination of bilberry extract and DHA in this study demonstrates promising results in controlling the progression of myopia in guinea pigs, making it a potential candidate for future therapeutic strategies. Both compounds appear to exert synergistic effects on ocular health through multiple mechanisms, including upregulation of Chrnb4 gene expression, enhancement of dopamine levels, and modulation of ECM remodeling. These findings are consistent with previous studies suggesting that natural bioactive compounds can play a key role in preventing or slowing the progression of myopia.

In clinical applications, the combination of DHA and bilberry extract could be explored as a non-invasive intervention for myopia management. Given their safety profiles and natural origins, these compounds could serve as a complementary treatment to conventional myopia control methods, such as optical devices or pharmaceutical agents. Further research is needed to confirm these findings in human studies and to evaluate the long-term efficacy and safety of such interventions.

## 6 Conclusion

In conclusion, the combination of bilberry extract and DHA offers a multi-targeted approach for controlling myopia progression. By regulating Chrnb4 expression, enhancing dopamine levels, and modulating the TGF-β/MMP-2/TIMP-1 signaling pathway, this intervention effectively prevents scleral remodeling and axial elongation. The results of this study provide valuable insights into the potential therapeutic use of natural bioactive compounds for myopia control and pave the way for the development of novel, non-invasive treatment strategies.

## 7 Limitations

This study also has several limitations. First, the experiments were confined to animal models, and whether the results can be directly extrapolated to the prevention and treatment of human myopia requires further validation. Second, parameters such as the dosage of bilberry extract and DHA, administration methods, and intervention duration used in the study may influence the experimental outcomes. Future research should explore the optimal treatment regimen.

## Data Availability

The raw data supporting the conclusions of this article will be made available by the corresponding author upon reasonable request, T-nL, lintn@fjmu.edu.cn.
